# A Digital Intervention for Primary Care Practitioners to Support Antidepressant Discontinuation (Advisor for Health Professionals): Development Study

**DOI:** 10.2196/25537

**Published:** 2021-07-16

**Authors:** Hannah Bowers, Tony Kendrick, Nadja van Ginneken, Marta Glowacka, Samantha Williams, Geraldine M Leydon, Carl May, Christopher Dowrick, Joanna Moncrieff, Chris F Johnson, Michael Moore, Rebecca Laine, Adam W A Geraghty

**Affiliations:** 1 Primary Care, Population Sciences and Medical Education University of Southampton Southampton United Kingdom; 2 Department of Primary Care and Mental Health University of Liverpool Liverpool United Kingdom; 3 Faculty of Health and Social Sciences Bournemouth University Bournemouth United Kingdom; 4 Department of Health Services Research and Policy London School of Hygiene and Tropical Medicine London United Kingdom; 5 Division of Psychiatry Faculty of Brain Sciences University College London London United Kingdom; 6 Pharmacy & Prescribing Support Unit Pharmacy Services NHS Greater Glasgow & Clyde Glasgow United Kingdom

**Keywords:** antidepressant discontinuation, intervention development, depression, primary care, digital intervention

## Abstract

**Background:**

The number of people receiving antidepressants has increased in the past 3 decades, mainly because of people staying on them longer. However, in many cases long-term treatment is not evidence based and risks increasing side effects. Additionally, prompting general practitioners (GPs) to review medication does not improve the rate of appropriate discontinuation. Therefore, GPs and other health professionals may need help to support patients discontinuing antidepressants in primary care.

**Objective:**

This study aims to develop a digital intervention to support practitioners in helping patients discontinue inappropriate long-term antidepressants (as part of a wider intervention package including a patient digital intervention and patient telephone support).

**Methods:**

A prototype digital intervention called Advisor for Health Professionals (ADvisor HP) was planned and developed using theory, evidence, and a person-based approach. The following elements informed development: a literature review and qualitative synthesis, an in-depth qualitative study, the development of guiding principles for design elements, and theoretical behavioral analyses. The intervention was then optimized through think-aloud qualitative interviews with health professionals while they were using the prototype intervention.

**Results:**

Think-aloud qualitative interviews with 19 health professionals suggested that the digital intervention contained useful information and was readily accessible to practitioners. The development work highlighted a need for further guidance on drug tapering schedules for practitioners and clarity about who is responsible for broaching the subject of discontinuation. Practitioners highlighted the need to have information in easily and quickly accessible formats because of time constraints in day-to-day practice. Some GPs felt that some information was already known to them but understood why this was included. Practitioners differed in their ideas about how they would use ADvisor HP in practice, with some preferring to read the resource in its entirety and others wanting to *dip in and out* as needed. Changes were made to the wording and structure of the intervention in response to the feedback provided.

**Conclusions:**

ADvisor HP is a digital intervention that has been developed using theory, evidence, and a person-based approach. The optimization work suggests that practitioners may find this tool to be useful in supporting the reduction of long-term antidepressant use. Further quantitative and qualitative evaluation through a randomized controlled trial is needed to examine the feasibility, effectiveness, and cost-effectiveness of the intervention.

## Introduction

### Background

Antidepressant prescribing has increased in England every year for more than 30 years [1], and Public Health England estimated that 4.5 million people (around 1 in 10 of the adult population) were taking an antidepressant in March 2018 [2]. This rise is mainly because of an increase in long-term use [3,4]—approximately 40% of antidepressant users have been on them for 2 years or more and 24% for at least 3 years [2].

Although long-term use of antidepressants is considered necessary in some cases to prevent relapse, surveys suggest that approximately one-third to a half of patients on long-term antidepressants have no evidence-based indication for continuing long-term use [5-7]. Attempts to discontinue antidepressants in these patients may improve their quality of life by avoiding adverse effects that tend to increase with longer-term use [8] and may also help to reduce the costs of prescriptions and consultations to the National Health Service [9].

Previous research has suggested that patients may continue to take antidepressants long term because of a lack of general practitioner’s (GP) review [10]; however, studies on prompting GPs to review patients on long-term antidepressants have resulted in only 6% to 8% of patients discontinuing, which did not significantly differ from usual care [11,12]. The barriers to stopping antidepressants are multifaceted, and thus, a complex approach to support withdrawal is needed, involving patients, primary care practitioners, and additional psychological support.

Qualitative studies on the long-term use of antidepressants have highlighted a number of barriers to discontinuation, such as the fear of relapse and beliefs about the necessity of long-term use [13]. Importantly, these studies have also highlighted the critical role of the GP-patient relationship and how this can help or hinder discontinuation. However, there is comparatively less research exploring the perspectives and experiences of health professionals (ie, GPs, nurses, pharmacists, and psychological practitioners) regarding antidepressant discontinuation. In a recent qualitative study, Bowers et al [14] highlighted key factors that served as barriers to GPs supporting patients to discontinue long-term antidepressants. These included uncertainty about the responsibility of initiating discussions about discontinuation, with GPs often suggesting it was the patients’ responsibility to start these conversations. Fear about destabilizing patients who were well was also a factor, alongside a perceived lack of information and support for safe discontinuation. Importantly, it may be possible to intervene to address such issues, thereby supporting successful discontinuation, where appropriate.

### Objectives

The REDUCE (Reviewing Long Term Antidepressant Use by Careful Monitoring in Everyday Practice) program aims to develop and trial safe, feasible, and effective ways to support antidepressant discontinuation. In this paper, we describe the methods involved in the development of a digital intervention aimed at changing the behavior of health professionals involved in antidepressant discontinuation as part of the REDUCE program. We then discuss how this method was implemented and the findings of the development work.

## Methods

### The Person-Based Approach

**Figure 1:**
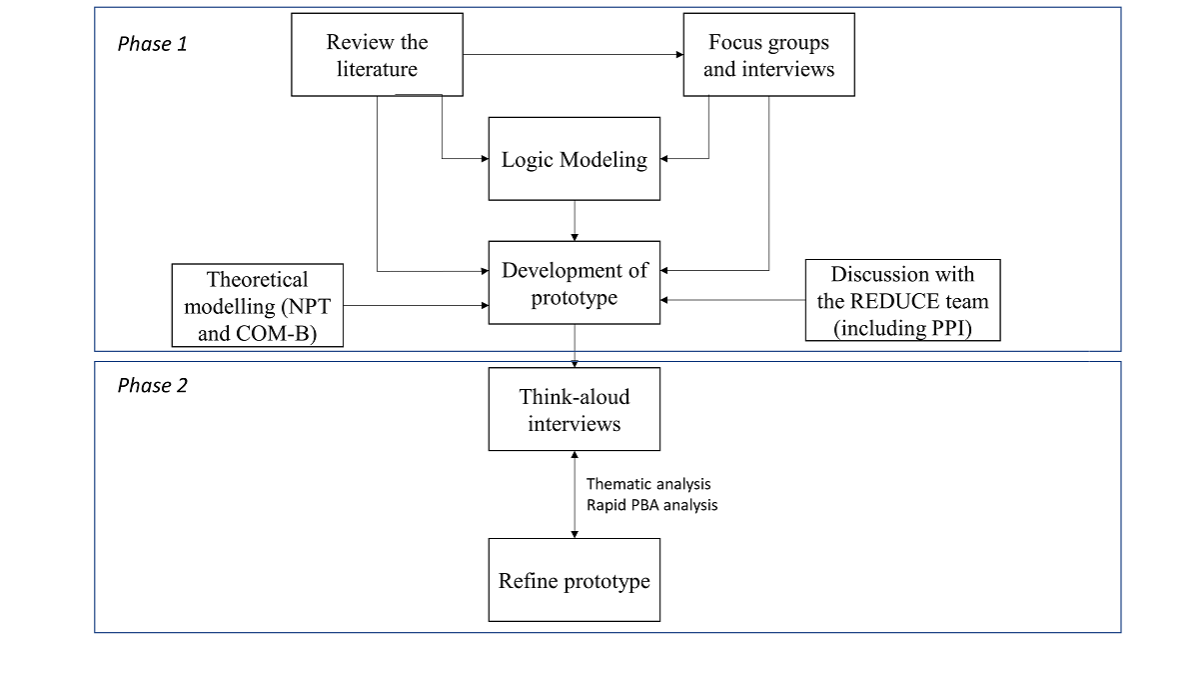
Intervention development method. COM-B: Capability, Opportunity, Motivation-Behavior; NPT: Normalization Process Theory; PBA: person-based approach; PPI: patient and public involvement; REDUCE: Reviewing Long Term Antidepressant Use by Careful Monitoring in Everyday Practice.

The mixed methods approach used to guide the development of the intervention for health care professionals takes commonly applied theory and evidence-based methods and integrates the person-based approach (PBA) [15]. The PBA is a systematic approach for integrating in-depth qualitative research across multiple stages of the development process. It involves the integration of open-ended qualitative interviews, qualitative meta-syntheses, and think-aloud qualitative optimization interviews with relevant theory and quantitative evidence base (Figure 1). Here, we will describe the key elements of this approach, including how we drew from theory (through behavioral analysis and logic modeling) and systematic reviews and conducted primary qualitative research to develop our intervention.

### Phase 1: Intervention Planning

#### Step 1: Systematic Reviewing

A qualitative thematic synthesis of 22 studies was conducted, as described in detail elsewhere [13]. In this thematic synthesis, our team identified barriers and facilitators to antidepressant discontinuation from both patient and health professional perspectives. Barriers and facilitators from health professionals’ perspectives were then used to inform the guiding principles (Textbox 1) and content for the intervention.

**Guiding principles for the intervention package: design objectives and key intervention features relevant to each design objective.**

**To persuade and inform health professionals about the reasons and benefits of discontinuing antidepressants**

- Ensure that the material covers patients’ motivations for stopping
- Include evidence that antidepressants can be withdrawn without harm

**To provide rapidly accessible information designed to increase self-efficacy for effectively managing discontinuation in patients**

- Simple, unobtrusive interface, using bulleted text as far as possible
- Offer a range of strategies for identifying and managing withdrawal effects
- Use of clear, visually mapped tapering schedules
- Acknowledge flexibility in working with patients

**To enable health professionals to support a wide range of patients and be used in a manner that best suits the health professionals**

- Provide content that will be relevant for patients in a wide variety of contexts
- Enable use in multiple ways (eg, as a linear educational system or as a resource that can be dipped in and out of), as and when the health professional deems necessary

#### Step 2: Primary Qualitative Research

We drew from our previously conducted qualitative study with health professionals regarding antidepressant discontinuation (refer to the study by Bowers et al [14] for full paper). This qualitative work comprised 4 focus groups and 3 individual interviews with 38 health care professionals. The focus groups and interviews explored views on long-term antidepressant use, negotiating the decision to discontinue with patients, their role in supporting patients, and important elements of the proposed interventions (including content for practitioners). Professionals included 21 GPs, 4 GP assistants, 7 nurse practitioners (NPs), and 6 mental health workers or psychological therapists. We used themes identified in the interviews and focus groups to inform the content and mode of presentation or delivery of the intervention.

#### Step 3: Development of Guiding Principles

As part of the PBA, guiding principles—a set of key assumptions followed throughout the process of the intervention development—were formulated. They included broad design objectives that inform how the core intervention strategies are applied and implemented with the aim to increase engagement [16]. The qualitive work (ie, focus groups, interviews, and qualitative metasynthesis) informed the formulation of the guiding principles for the health professional intervention.

#### Step 4: Behavioral Analysis and Logic Modeling

We drew on implementation and behavioral theory in addition to our qualitative work and discussions with experts (including patient representatives, GPs, psychiatrists, psychologists, sociologists, and health service researchers) to identify key components of the intervention. We conducted a behavioral diagnosis using the Behavior Change Wheel (BCW) and the COM-B Model (capability, opportunity, motivation and behavior) of behavior change [17,18] to identify key behaviors that needed to be addressed or targeted in the intervention and their determinants or context. Our proposed ideas about intervention content were then mapped theoretically using the BCW and Normalization Process Theory (NPT) [19]. NPT is a framework for understanding the key mechanisms that shape implementation processes. It focuses on the work that participants do to incorporate components of complex interventions into their everyday lives [19]. In addition, we used logic modeling to draft how the intervention may work to effectively support appropriate discontinuation. Logic models [20] represent theories of change in a diagrammatic form. They are a useful tool to highlight assumptions regarding potentially important mechanisms and are adapted as data become available.

#### Step 5: Drafting Intervention Content

The intervention’s content was developed using findings from the qualitative metasynthesis of the literature, primary qualitative work, behavioral analysis, and logic modeling. Written intervention content was drafted by a member of the content development team (HB) using what was learned from the qualitative work and theoretical modeling. These drafts were then discussed by the content development team (HB, AG, and MG) and then the wider team (which included patients, GPs, sociologists, psychologists, and psychiatrists). Discussions led to iterations of the content until the team was in agreement. This process resulted in a prototype intervention that addressed key barriers and facilitators identified in the primary qualitative work and metasynthesis and that fit with the guiding principles, behavioral analysis, and logic model.

The content was transferred into web-based pages in LifeGuide (a software program for developing web-based interventions [21]), and further discussions and iterations took place, modifying the presentation of information, before reaching an agreement on the prototype to be used in the intervention optimization phase.

### Phase 2: Intervention Optimization

#### Design

Following the PBA, think-aloud qualitative studies were used to optimize a digital prototype of an intervention [15]. They are designed to explore users’ perspectives on the intervention’s content and its relevance, appropriateness, and understanding as well as users’ views on functionality, usability, and design of an intervention. Ethical approval for the study was granted by the National Health Service South Central Oxford B Research Ethics Committee. Participants provided informed consent and were identified only by participant ID.

#### Participants

Participants were health professionals from primary care practices in the south of England who responded to a call from the local National Institute for Health Research (NIHR) clinical research network Wessex for health practitioners to participate in a semistructured face-to-face interview. The study team then contacted interested participants and arranged to meet them at their home or at their practice.

#### Procedure

Eligible participants met with researchers (HB, TK, RL, and/or SW) who invited them to engage with the digital prototype intervention and say what they were thinking aloud. When necessary, the interviewer prompted participants (eg, asking practitioners, “How do you feel about the information on this page?”). Interviews ranged from 32 to 82 minutes in length and were audio recorded. The interview schedule is presented in Multimedia Appendix 1.

Following each interview, comments were discussed among the study team. Comments were initially discussed based on notes from the interviews, and further discussions were conducted based on the analyzed transcriptions. This was done to modify the intervention in a timely manner. As a result of these discussions, amendments were made to the intervention in an iterative fashion; 4 subsequent intervention versions were shown to the participants following the first prototype. Participants participated in only 1 interview. Therefore, participants who were recruited later in the study saw an updated and amended version of the intervention, different from the versions seen by participants recruited earlier. This process allowed the changes that were made as a result of practitioner feedback to be shown and discussed with practitioners recruited later in the study. Interviews with practitioners continued until data saturation was reached, that is, when comments about the intervention reflected that no further changes were necessary and there were, therefore, no new codes identified.

#### Analysis

Interviews were transcribed verbatim and analyzed using 2 analytical methods. The first method comes from the PBA and is more rapid than thematic analysis, involving the use of coding tables specifically designed to highlight positive and negative comments about the intervention. All minor amendments to the intervention were agreed upon following discussion with the intervention development team. Issues that were likely to affect participant engagement or intervention effectiveness were discussed with the broader group before making changes. Along with positive and negative comment tabulation, we also conducted a thematic analysis of general or overall views about the intervention using NVivo software (QSR International) [22,23]. This more in-depth analysis aimed to capture participants’ views of the intervention and ideas about how they may engage with it. The thematic analysis was central in making amendments to the intervention. HB independently coded the transcripts using thematic analysis and discussed an initial coding frame with a second researcher (AG). The broader team was included in discussions about labeling and interpretation. The findings of the in-depth thematic analysis are presented here.

## Results

### Phase 1: Intervention Planning and Development

#### Step 1: Systematic Reviewing

Our qualitative thematic synthesis identified key barriers and facilitators to discontinuation for both patients and health professionals (refer to the study by Maund et al [13] for full details). The following issues were regarded as key to inform the development of the health professional intervention:

- GPs reported patient dependence on antidepressants and patients’ difficult life circumstances as barriers to discontinuing antidepressants.
- Patients perceived their doctor as the navigator of discontinuation, responsible for recommending or approving the decision to discontinue, and responsible for initiating the discussion.
- Patients expected their doctor to support and guide them through the discontinuation process.
- Both patients and GPs acknowledged that the GP often does not have the time necessary to best support discontinuation.

The findings from this thematic synthesis informed the guiding principles, behavioral analysis, and logic model, which formed the basis for intervention content selection and development.

#### Step 2: Primary Qualitative Research

Thematic analysis [22] of the focus groups and interviews resulted in 5 themes exploring barriers and facilitators for health professionals initiating and discussing discontinuation with patients (refer to the study by Bowers et al [14] for further details of the findings). After discussion, the following issues were determined to be the most relevant and directly informed intervention development:

- When neither the health professional nor the patient raises the topic of discontinuation, this may result in a form of collusion where both parties are assuming that the other party wants antidepressant treatment to continue.
- Health professionals were concerned about destabilizing well patients—some felt that continuing antidepressants was easier and less risky.
- Knowing the patient’s history and past experiences with depression and antidepressants facilitated discontinuation discussions, although poor continuity made such conversations less likely.
- Insufficient information and understanding of antidepressant discontinuation made them less confident in their ability to successfully taper patients off antidepressants.
- Conversations about discontinuation are time consuming and require follow-up appointments, which are often limited.

These literature syntheses and qualitative interview findings informed the development of guiding principles, a behavioral analysis, the logic model, and the intervention content. For example, understanding that the GP’s time constraints can be a barrier resulted in a key design objective being *to provide rapidly accessible information designed to increase self-efficacy for effectively managing discontinuation in patients* (Textbox 1). The qualitative findings also directly informed the content of the intervention. For example, recognizing the collusion that results in both parties assuming that the other wants treatment to continue led to an intervention module dedicated to *broaching the subject* (Textbox 2).

**Outline of the intervention content.**

**Content and Description**

- Why reduce
  A rationale for discontinuation highlighting patients’ views on antidepressants and why they would prefer not to take them and covering evidence on relapse rates for patients discontinuing in primary care
- Broaching the subject
  Guidance on addressing the topic of discontinuation within a consultation and acknowledging time constraints
- When to start tapering
  Information about which patients might be appropriately considered for discontinuation and what time might be most appropriate to initiate the withdrawal
- Reduction schedules
  Suggested reduction schedules for different medications under different circumstances (eg, depending on drug half-life or patients’ previous experiences with withdrawal)
- Dealing with withdrawal symptoms
  Information about distinguishing relapse from withdrawal symptoms and how to respond to mild, moderate, and severe withdrawal symptoms
- Dealing with relapse
  Information about how patients are advised to manage their relapse prevention
  Guidance on when it might be advisable to reinstate antidepressants in the face of relapse
- ADvisor for Health Professionals for patients
  A brief overview of the content covered in the patient digital intervention to enable practitioners to recommend information in the web-based resource for patients to look at home
- Printable pages
  Printable handouts to give to patients (eg, a tapering regime template)
- Resources
  Links to relevant papers and guidelines related to antidepressant discontinuation

#### Step 3: Guiding Principles for the REDUCE Intervention Package

These principles included design objectives and design features (Textbox 1). Our 2 broad design objectives were (1) to build confidence that discontinuing antidepressant medication is safe and achievable in the long term and (2) to be an accessible, motivational resource that supports patients in managing their withdrawal, which aligns with their preferences. These objectives were achieved through the development of the patient digital intervention (which is described elsewhere [24]) and through the health professional intervention (eg, patients’ views on enabling empathy and GP support informed the section for GPs to build their confidence that discontinuation is safe). The design features that support these objectives are presented in Textbox 1.

#### Step 4: Behavioral Analysis and Logic Modeling

The behavioral diagnosis for the health professional intervention can be found in Multimedia Appendix 2 [17,18]. Reducing and stopping antidepressant medication was our target behavior, and reflective motivation and psychological capability were considered the key constructs for changing this behavior. In terms of increasing the psychological capability of health professionals, this would involve improving knowledge about discontinuation and setting expectations around the withdrawal process to build self-efficacy in discontinuing antidepressants. To improve reflective motivation for health professionals, the intervention targeted beliefs about treatment that may act as barriers to discontinuation (eg, that withdrawal is always challenging and unachievable).

**Figure 2:**
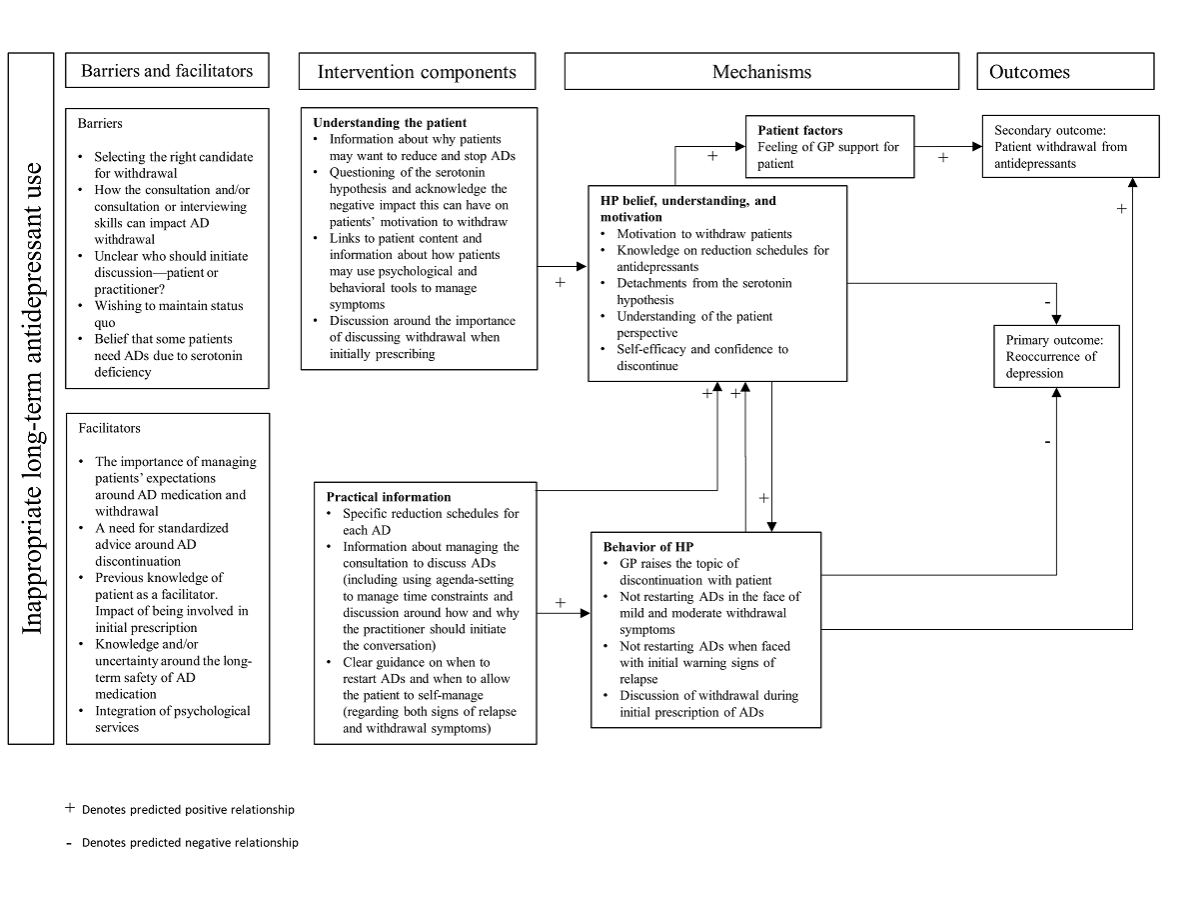
Logic model for Advisor HP. AD: antidepressant; GP: general practitioner; HP: health practitioner.

Content for particular intervention modules was drafted and mapped against constructs from the BCW and NPT (Multimedia Appendix 3). For example, in the module dedicated to providing a rationale for discontinuing antidepressants, the key BCW construct addressed is reflective motivation, and for NPT, the key constructs are enrollment and systematization. The logic model for the intervention is shown in Figure 2. Depicted in the logic model are the hypothesized relationships between barriers and facilitators to discontinuation intervention components, mechanisms, and outcomes.

#### Step 5: Drafting Intervention Content

A prototype digital web-based intervention was developed for health professionals involved in supporting patients to discontinue long-term antidepressant use. The contents of the web-based intervention are described in Textbox 2. A digital intervention for patients was also developed, as reported separately [24].

The intervention was designed to be flexible to cater to the time constraints under which GPs work. As such, the topics covered were optional and accessible from the main menu page; users could skip content that they felt would not be beneficial to them. This also meant that necessary information could be accessed quickly during a consultation.

### Phase 2: Intervention Optimization

#### Participants

Of the 32 practitioners who expressed interest, 13 could not be reached by the study team or could not take part (eg, because of time constraints or sickness), resulting in a final sample of 19 practitioners (2 NPs and 17 GPs); there were 8 men and 11 women. The mean age was 44.29 (SD 7.13) years, and the mean number of years since qualification was 14.39 (SD 7.77) years (Table 1).

**Table 1:** Demographic information about think-aloud interview participants.

ID	Sex	Role	Years qualified	Iteration seen
GP^a^/15/01	m^b^	GP	4	1
GP/11/01	f^c^	GP	22	1
GP/11/02	f	GP	32	1
GP/13/01	m	GP	14	3
GP/14/01	m	GP	34	1
GP/16/01	f	GP	14	2
GP/17/01	f	GP	27	2
GP/18/01	f	GP	26	2
GP/08/01	m	GP	20	1
GP/19/01	m	GP	21	3
NP^d^/08/01	f	NP	17	1
GP/21/01	f	GP	5	4
GP/22/01	f	GP	16	4
GP/21/02	f	GP	8	5
GP/22/02	m	GP	Not recorded	4
NP/23/01	f	NP	Not recorded	5
GP/25/01	m	GP	12	4
GP/27/01	m	GP	23	5
GP/27/03	f	GP	13	5

^a^GP: general practitioner.

^b^m: male

^c^f: female.

^d^NP: nurse practitioner.

#### Findings

##### Summary of Thematic Analysis

The thematic analysis of the 19 transcripts resulted in the following five themes: how Advisor for Health Professionals (ADvisor HP) would be used in practice, pitching at the right level for health professionals, *ADvisor HP is evidence based*, the need for brevity and quick access, and *ADvisor HP is useful*. Participant identifiers are presented after each quote, and the iteration number refers to the iteration of the intervention that the practitioner saw (ie, iteration 1 means that the participant saw the first iteration of the intervention). The changes made at each iteration are listed in Textbox 3.

**Examples of changes made at each iteration based on the feedback from the think-aloud interview.**

**Changes made following iteration 1 feedback**

- Clarified reporting of statistics on relapse rates after discontinuation
- Clarified that side effects improve once the antidepressant is stopped
- Provided further clarity around antidepressant use and discontinuation for treating anxiety
- Added qualifying statements such as “experienced GPs will know” to address comments around some of the information being well-known to more experienced general practitioners (GPs) but necessary for newly qualified GPs and nurse practitioners
- Added that there is evidence to support that asking about additional concerns in a consultation does not extend the length of a consultation
- Amended the definition of patients who are currently well to include those who may still have mild symptoms
- Emphasized that the included reduction schedules are suggestions and may be individualized
- Fixed typos
- Added clarity on the timeframe of withdrawal symptoms compared with symptoms of relapse
- Provided specific information about anxiety as a withdrawal symptom

**Changes made following iteration 2 feedback**

- Revised text to be more concise, using more bullet points
- Changed navigation, so that each module provides an overview with an option to read about it in more detail
- Revised tone to be less prescriptive

**Changes made following iteration 3 feedback**

- Formatting changes (eg, font choices, text box color, and image layout)
- Navigation changes (eg, making the end of a section and navigation back to the main menu clearer)
- Correction of typos

**Changes made following iteration 4 feedback**

- Navigation buttons amended (ie, removing “exit” buttons to help people navigate back to the main menu)
- Added clarity around what information patients get from their digital intervention
- Revised wording around the timing of tapering regimes

##### How ADvisor HP Would Be Used in Practice

Participants described using ADvisor HP as a resource of information which they can refer to. Nine participants felt that they would first read through ADvisor HP as a whole and that once all of the information was read, they would not need to refer back to the same information many times:

I really like it. Is it potentially going to be — I mean — not quite a one-off visit...but, you know, if you had worked all the way through it, yes, you don’t have a photographic memory, but — because you’ve given us some nice, simplistic regimes, it isn’t something necessarily that I’m going to refer to umpteen times a day [I: Right], because, of course, once you’ve done it three or four times, you’re going to know what that regime is.

GP/22/01, iteration 4

Many health professionals liked that they could engage with the intervention in different ways, for example, *dipping in and out* (n=4) and using in a targeted fashion for specific knowledge, as needed (n=7). Two participants said they would save a shortcut on their computer for easy and quick access to information if and when they needed it:

I do like that you pick what you want to read and so you can dip in with what you think you want to know and dip out. It’s your choice what you do; you don’t have to read through the whole thing to get to what you’re particularly interested in, because there will be times when you think — actually I just don’t know what to do about — about that, so you can dip in and look at that. So, yes, I like that.

GP/11/02, iteration 1

Three health professionals (2 nurses and 1 GP) talked about using ADvisor HP more regularly and making it a part of everyday practice:

Yes, I would definitely use it in practice — on a day-to-day — like I said, just looking in between consultations or if I had a particular patient that I’d discussed it with and — I may even kind of ring them back if I’d looked at it after the consultation and make suggestions.

NP/08/01, iteration 1

Some health professionals talked about printing copies of the information both for easy access for themselves and to hand to patients, as they felt there was something beneficial in the act of physically giving something. Therefore, we added 2 printouts to be given to patients—one to direct patients to specific content in the patient intervention, ADvisor, and another to be used to provide a personalized tapering regime:

Patients like to go out the room holding something, don’t they, whether it’s a prescription or something and to give them something to do for themselves, gives them a bit more ownership of it and it feels like they’re doing something for themselves, rather than having to rely on us and the tablets all the time.

GP/18/01, iteration 2

Very few health professionals felt that they would use ADvisor HP within a consultation for their own information. However, some talked about using ADvisor HP collaboratively with a patient within a consultation as a way of showing the patient reliable information and providing evidence for their clinical decision making:

I think sometimes, although it’s for the doctor, sometimes it’s quite useful to show the patients these things as well. I don’t tend to use the internet that much but sometimes when you get a leaflet or you find something that’s kind of - and to us, if you show it to the patients as well, then it builds up that trust a bit more as well, that that’s why you’re doing it. You show evidence for things.

GP/18/01, iteration 2

When discussing how they envisage ADvisor HP being used in practice, 3 GPs reported that they did not feel they would use it. The reasons they provided were that it would be difficult to find the time to read it and that they already knew the information contained in ADvisor HP:

I think most GPs would love to get their patients off antidepressants; if we could give patients a link to a website supporting that, then that’s great and we could come to a plan together, but I don’t know that I necessarily need to read that, to make that plan.

GP/16/01, iteration 2

There was acknowledgment among some health professionals that work would need to be done to get other HPs to engage with and be aware of ADvisor HP:

I think it would be really helpful. I’m not quite sure how you’re going to — market it as such, because obviously all of us get absolutely inundated with — there’s a new tool for this and a new resource for that and new guidance on this and — we have to cherry pick what we actually even — bother looking at, let alone going through.

GP/22/01, iteration 4

##### Pitching It at the Right Level for GPs

Later iterations reduced the detail of information where health professionals highlighted that it was common knowledge or unnecessary and it was stated in ADvisor HP that information may not be new but may act as a reminder (Textbox 3). Two GPs also commented that the tone of the intervention could be less prescriptive:

So in, under this talking to the patient heading, sounds like a lot of it is so obvious that it’s patronising and could be annoying to a GP.

GP/15/01, iteration 1

Some of these health professionals explained that it was still useful to include information they already knew, as it can act as a reminder and may be useful to less experienced practitioners and trainees. A GP explained that it is reassuring to read that the guidance corresponds to what they are already doing in practice:

I suppose a lot of it — you know — I feel I know already, in truth, but there’s — it’s a reminder. And actually, you know, it’s a prompt that when — when I’m next speaking to somebody, I might just — and we’re talking about coming off antidepressants, I might actually just specifically touch on those areas, whereas before I would have probably waited until they told me those things.

GP/27/01, iteration 5

A GP explained that information that may seem obvious to some GPs does not need to be excluded from ADvisor HP. By having a main menu and easy access to different sections, one could quickly move from one section to another if they felt that the information was not something they needed to read:

If you go on to a section and you think, I know that already, you could always come out of it.

GP/11/02, iteration 1

Despite these comments, many of the GPs reported learning something new in ADvisor HP, in particular, new information about antidepressants (eg, switching to another antidepressant to help avoid distressing withdrawal), patient perspectives (eg, patients expect their GP to initiate discontinuation conversations), and how psychological therapeutic techniques may help patients to discontinue antidepressants:

That’s interesting. It says many patients may continue taking their antidepressant because they believe the GP wants them to, because I’ve always thought it was almost the opposite way around, that patients want to carry on their antidepressants and GPs are trying to take them off it. And I often find that people are saying — please don’t make me stop them and wanting to carry on with them for longer, but probably I’m wrong; maybe that’s just an anecdotal thing for me and it seems, from this, that they’ve shown that if a GP doesn’t suggest discontinuing, that they’ll just carry on and on and on.

GP/27/03, iteration 5

Two GPs explicitly stated that they would change their practice following new information in ADvisor HP. They would now be more inclined to broach the subject of stopping, more likely to ask patients about upcoming life events that may interfere and be less inclined to restart antidepressants in the face of withdrawal symptoms:

So if that [symptoms of depression or anxiety] happens within a couple of days of the dose change then it’s probably not a relapse; it’s probably more a withdrawal. But it’s a good reminder for me, because I think if I had a patient come back to me, having cut their SSRI, then within a few days was saying they felt awful, I might just be tempted to put them back on it, really. [I: Right] Whereas actually now I might be tempted to — encourage them to stay with it for a bit longer.

GP/27/01, iteration 5

##### ADvisor HP is Evidence Based

Health professionals reported reassurance that the information was in line with the National Institute for Health and Care Excellence (NICE) guidance and liked the references to research to support the information in ADvisor HP:

So I guess it’s reassuring to have some evidence-base and some NICE guidelines incorporated. As this is a practitioner website, I guess it — it’s helpful to give peace of mind and reassure people that actually it’s the right thing to do, to come off when — when certain criteria have been fulfilled, as it were

GP/08/01, iteration 1

In particular, evidence showing the relatively low relapse rates of patients was found to be reassuring by 5 of the GPs in the sample. They also felt that these figures would be useful in reassuring the patient, who may fear discontinuation:

I actually — I’m quite impressed with the number that came off; that’s more than I thought. [I: Okay] So that’s quite good and it probably would make me a bit more motivated to try and get people off.

GP/16/02, iteration 2

Also, 2 GPs acknowledged that the existing guidelines are not easy to navigate when looking for particular pieces of information or guidance. They felt that ADvisor HP made it easy to access the information needed:

So I think a lot of people would be interested in the research evidence for it. Yes, just looking who is eligible to discontinue, which I think is quite important, because NICE guidelines tend to be a little bit thick and long winded and it’s hard to really condense them, I think, to get the information you need, sometimes, so that’s quite helpful, having just those several bullets points there.

GP/25/01, iteration 4

##### The Need for Brevity

Many health professionals highlighted their strict time constraints and, thus, the need to access information quickly. Five GPs commented, particularly in the earlier iterations, that ADvisor HP was time consuming to go through and that this may prevent them from being able to use the intervention. A large number of health professionals reported that there was more information than necessary. Some suggested that although the content was useful, it could be presented more concisely:

I think the overwhelming thing is probably we have such limited time and we need to be able to look at everything so quickly and you need to be able to find something straightaway. So I think it is, because you very much go straight into it [the main menu] and then you go to Reduction Schedules, as long as you’ve looked at it before and know where they are.

GP/21/01, iteration 4

However, 4 GPs and 1 NP reported that the intervention was concise and not too big to go through. Nevertheless, in further iterations of ADvisor HP, the content was revised to be more concise. It is possible that these changes resulted in health professionals finding the content more manageable to go through:

I like the type font, I think it’s quite clear, the photos are appropriate. It’s very easy to read. The whole package isn’t too big and you know what you’re dealing with.

GP/21/02, iteration 5

##### ADvisor HP Is Useful

Throughout the interviews, GPs and nurses commented that the information in ADvisor HP was useful. There were mixed views, in that different pieces of information were reported as useful by different participants. This fits with the guiding principles of ADvisor HP, in that the information should be flexible so that participants can access what they find useful, without the need to view the information they may not wish to view at that time. Tapering regimes were highlighted as particularly useful by 13 of the 15 health professionals, which fits with our primary qualitative evidence that there is a lack of existing clear, accessible guidance:

I think the most helpful bits are probably actually looking at the advice on the medications and the exact dosage regimes to use and I think probably the information for the patients is all quite good.

GP/21/01, iteration 4

Two GPs did not find tapering guidance useful, reporting that they already knew it and that drug regimens need tailoring to each individual patient.

Many health professionals also reported that the information on how to deal with withdrawal symptoms and how to distinguish between them and signs of relapse was useful:

Yes, the contrast between withdrawal and relapse, especially the timeframe.... Yes, I think that’s quite a good table; I like that

GP/16/01, iteration 2

By the end of the interviews, the main issues with ADvisor HP were resolved. The intervention was deemed ready for the forthcoming feasibility trial (which will be reported elsewhere).

## Discussion

### Principal Findings

Using the PBA, we developed a digital intervention designed to support health care professionals helping patients to withdraw from long-term antidepressant treatment. This intervention was based on a systematic literature review, in-depth qualitative research, and discussion with experts. The intervention aims to provide easily accessible, evidence-based guidance for health professionals and is grounded in practitioner perspectives and preferences. It was designed to be part of an intervention package along with a patient digital resource and patient telephone support with a psychological practitioner.

The think-aloud interview findings suggest that ADvisor HP could work in practice, as the information is quickly accessible and based on the latest evidence. The intervention will be examined in a feasibility randomized control trial in which a qualitative and quantitative process evaluation will be carried out. This feasibility study will explore how practitioners engage with the intervention to support to patients discontinuing antidepressants. Further intervention modifications may occur in response to the findings of the feasibility trial, in line with the latter stages of the PBA [15].

A digital intervention may be particularly useful in imparting knowledge and possibly more limited in changing practitioners’ attitudes and developing their skills. Educational research suggests that attitudes toward mental health problems may need to be challenged in peer group discussions and that a combination of modeling, role-play, and video feedback might be needed for the acquisition of new skills [25]. However, such educational initiatives are expensive and time consuming, and given the high prevalence of inappropriate long-term prescription of antidepressants and the time pressures under which primary care practitioners work, interventions that can quickly be applied at scale are needed.

Many previously developed tools to support practitioners in deprescribing have focused on clinical decision-making support systems or polypharmacy, particularly in older adult patients [26,27]. These tools often result in increased deprescribing; however, our qualitative work suggests that further education and belief or attitude change around antidepressant discontinuation would be needed to support deprescribing for long-term antidepressants. A study of antibiotic prescribing provided GPs with an internet education tool that was optimized using think-aloud methods [28]. This intervention resulted in significantly lower rates of antibiotic prescribing [29]. In using the PBA to develop ADvisor HP, health professionals’ beliefs have informed the initial development and the optimization of the intervention, which may therefore support belief and behavior change.

### Limitations

This study has some limitations. Many of the practitioners recruited for the focus groups and think-aloud interviews were recruited from less deprived regions of the south of England. Many patients who live in these regions are from more affluent backgrounds, with few from ethnic minorities. Therefore, the perspectives in this work may not represent broader cultures and experiences of antidepressant treatment nationally. In the feasibility work and the subsequent fully powered randomized control trial, further qualitative work will be carried out with a larger and more diverse population of practitioners in England and Wales.

It is possible that the practitioners who were interested in participating were already interested in mental health research and may, therefore, be more knowledgeable than other practitioners who may show a less keen interest in this research topic. The potential bias in our qualitative sample may explain why some GPs felt that the information was not new. Some GPs who participated in the think-aloud interviews felt that they did not need the information in the intervention, but nevertheless, they thought that it would be useful for GP trainees and doctors who are relatively new to UK family practice. Recruiting practices in different regions with a more diverse sample in the feasibility and main trials may encourage participating practitioners who are less informed about antidepressant discontinuation to take part. This development work included only 2 NPs, of whom both were prescribers responsible for managing long-term antidepressant use in primary care. Many practices do not involve NPs in the management and discontinuation of antidepressants. This limited representation of NPs made it difficult to identify differences between GPs’ perspectives and nurses’ perspectives. Conducting more research with NPs and other health care professionals who may be involved in antidepressant discontinuation would be useful to further understand how this intervention may be used. Exploring views on the intervention with a broader sample will help ensure that the intervention is pitched at the right level.

### Conclusions

This development work has demonstrated the need for a brief and accessible intervention for health professionals. In addition, the think-aloud interviews suggested that the developed intervention may contribute to a change in attitudes and behaviors related to antidepressant discontinuation. The randomized trial will test whether the intervention works in terms of enhancing the discontinuation of antidepressants, while measuring the effects on depressive symptoms.

## Appendix

Multimedia Appendix 1 Interview schedule.

Multimedia Appendix 2 Behavioral diagnosis.

Multimedia Appendix 3 Theoretical modeling for intervention content.

## Abbreviations

ADvisor HP: Advisor for Health Professionals
BCW: Behavior Change Wheel
GP: general practitioner
NICE: National Institute for Health and Care Excellence
NIHR: National Institute of Health Research
NP: nurse practitioner
NPT: normalization process theory
PBA: person-based approach
REDUCE: Reviewing Long Term Antidepressant Use by Careful Monitoring in Everyday Practice
