# Supplementary material for: A Digital Intervention for Primary Care Practitioners to Support Antidepressant Discontinuation (Advisor for Health Professionals): Development Study
Source: J Med Internet Res. 2021 Jul 16;23(7):e25537. doi: 10.2196/25537 (PMC8325079; doi:10.2196/25537)
Supplement: Multimedia Appendix 1 [file jmir_v23i7e25537_app1.docx]

**
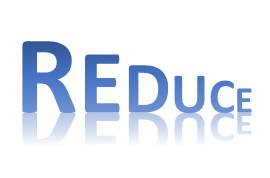
**
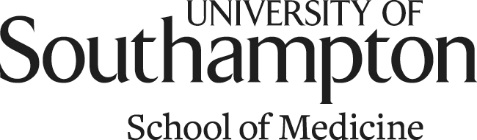


**REDUCE Study Workstream (WS) 3: REviewing long-term anti-Depressant treatment Use by Careful monitoring in Everyday practice**

**THINK-ALOUD INTERVIEW SCHEDULE WITH PRACTITIONERS**

*Below is a list of topics/questions to be discussed in this study. The qualitative work will remain flexible with respect to participants’ agendas but we will cover the broad topics/questions noted. It is common in qualitative work to iteratively develop topics and questions as new ideas emerge from early data collection. Therefore, we may add new topics as the interviews progress and data collection continues. However, the key topics of exploring participants’ views of the prototype intervention will remain the same.*

**Introduction**

1. Re-introduce self and purpose of interview
2. Check with participant:

• That they are still willing to be interviewed, and to be audio recorded

• Remind them it will take approximately 60-90 minutes

• That they are comfortable in a quiet place where they will not be disturbed

1. Remind participant that:

- Their responses will be kept confidential, and quotes used in the results will not identify them as an individual;
- They can change their mind about taking part in the study and stop the interview at any point.

1. Remind participant that the study will ask them to look at the online intervention and use it as they normally would, but they will be asked to say everything they are thinking aloud. They will also be asked questions about the intervention. Remind participants: There are no right or wrong answers as it is your views that are important to us.
2. Ask if the participant has any questions.
3. Start recording.

**Section 1: Think-aloud and researcher prompts**

Explain to them that you want them to look at the website and use it as they normally would, but say everything that they are thinking out loud. Tell them that you will remind them to do this so that they don't forget as it’s very easy to forget! If you think it would help then get them to try counting the windows in their house whilst saying everything that they are thinking out loud.

Possible prompts:

- What are your first impressions of this page?
- What are you thinking now?
- What made you choose that option?
- What do you think about [this activity, this information]?
- Can you tell me a bit more about that?
- What is it you like about that?

**Section 2: Post-think-aloud questions**

- Overall, what do you think about AD-visor?
- Can you tell me about anything that you liked about the website?
- Was there anything that you found surprising in AD-visor?
- Can you tell me anything about the website that you were less keen on?
- Can you tell me about anything that you think should be changed?
- Is there any information that you feel is unnecessary?
- Is there any information you would like to see that was not included?
- If you were supporting a patient withdrawing from your antidepressants, which parts of AD-visor do you think you would like to look at and why? (E.g. dealing with withdrawal symptoms, tapering schedules, how to broach the subject etc.).
- How might you see AD-visor fitting into practice? (E.g. would you use it in or outside of consultations? What might prevent you from using it in practice?)

***Anything wish to raise that hasn’t been discussed?***

***Any questions?***

***Moderator to check with observer for any further questions, then close Interview.***

**Debrief:**

- Tell participants audio recorder is now being switched off.
- Thank participants for taking part in the interview; excellent discussion.
- Revisit consent and reply slip.
- Ask if the participants have any questions / offer opportunity to discuss further following interview.
- Let participants know will be sending summary of results at the end of the study.
- Distribute travel claims and inform invoices will be sent to practice ASAP.
- Thank participants again for taking part.
