# Supplementary material for: A Digital Intervention for Primary Care Practitioners to Support Antidepressant Discontinuation (Advisor for Health Professionals): Development Study
Source: J Med Internet Res. 2021 Jul 16;23(7):e25537. doi: 10.2196/25537 (PMC8325079; doi:10.2196/25537)
Supplement: Multimedia Appendix 2 [file jmir_v23i7e25537_app2.docx]

**Appendix B – Behavioural Diagnosis**

| **Target behaviour: Reducing and stopping antidepressant medication** | | |
| --- | --- | --- |
| **BCW/COM-B Components** | **What needs to happen for the target behaviour to occur?** | **Proposed intervention element** |
| **Physical capability**  *Physical skill, strength or stamina* | - Understanding how to reduce doses physically | - **Internet intervention modules: tapering regimes** |
| **Psychological capability**  *Knowledge or psychological skills, strength or stamina to engage in necessary mental processes* | - Belief and confidence in one’s ability to manage discontinuation   *Social Cognitive Theory (SCT) and research will be broadly drawn on to ensure information/techniques are described and applied to align with evidence-based principles for increasing self-efficacy* | - **Internet intervention modules: accessible guidance on how to manage tapering, withdrawal and relapse** |
| **Physical opportunity**  *Opportunity afforded by the environment involving time recourses, locations, cues, physical* *affordance* | - Ability to access the information when needed | - **Information which is accessible from a Main Menu with direct links to useful information** |
| **Social opportunity**  *Opportunity afforded by interpersonal influences, social cues and cultural norms that influence the way we think about things* | - Clarity around who is responsible for broaching the subject of stopping | - **Internet intervention modules: Broaching the Subject of Stopping** |
| **Reflective motivation**  *Reflective processes involving evaluations/beliefs about what is good and bad, and plans (self-conscious intentions)* | - Reassurance that stopping is something a patient can try. - Understanding around who is eligible to try tapering and when to begin discontinue   *Inductive qualitative work (meta-synthesis and primary qualitative research) and theory will be used to inform this material* | - **Internet intervention modules: Why reduce, When to Start Tapering** |
| **Automatic motivation**  *Automatic processes involving emotional reactions, desires (wants and needs) impulses, inhibitions, drive states and reflex responses* | - Reduce fear around patient relapse | - **Internet intervention modules: include evidence on the likelihood of relapse, patient outcomes and how to manage relapse** |
| **Behavioural diagnosis of the relevant COM-B components** | Although all areas of the COM-B model will need to be addressed to some extent, **psychological capability** and **reflective motivation** are likely to be the key targets for a supported digital intervention to help practitioners support patients who are withdrawing from antidepressant medication |  |

**References:**

1. Michie S, van Stralen MM, West R. The behaviour change wheel: a new method for characterising and designing behaviour change interventions. Implement Sci. 2011;6:42.

2. Michie SF, Atkins L, West R. The behaviour change wheel: a guide to designing interventions. London: Silverback Publishing; 2015
