# Supplementary material for: A Digital Intervention for Primary Care Practitioners to Support Antidepressant Discontinuation (Advisor for Health Professionals): Development Study
Source: J Med Internet Res. 2021 Jul 16;23(7):e25537. doi: 10.2196/25537 (PMC8325079; doi:10.2196/25537)
Supplement: Multimedia Appendix 3 [file jmir_v23i7e25537_app3.docx]

| Intervention module | Page | Content | Evidence | NPT construct | BCW construct |
| --- | --- | --- | --- | --- | --- |
| Why reduce and discontinue antidepressants? | Who is eligible to discontinue? | A bullet point list of criteria a patient should meet to be considered eligible to discontinue treatment, based on NICE guidelines. | During focus groups, practitioners reported wanting more information about assessing who would be suitable to discontinue and that they would like information from existing guidelines to be presented more clearly and accessibly. | Individual specification | Psychological capability |
|  | Why reduce and stop antidepressants? | Provides a rationale for discontinuation by highlighting that many patients would rather not take antidepressants if they can stay well, and that many people won’t relapse if they discontinue | Using normalisation process theory, primary qualitative work with health professionals indicates that evidence that the intervention can benefit patients is important with regards to encouraging the practitioners to engage with the intervention. | Enrolment, systematization, Internalisation | Reflective motivation |
|  | Patient problems from taking antidepressants long-term | Highlights the problems encountered by patients such as side effects, which worsen with the length of treatment | Primary qualitative work with practitioners indicates that they would like information about the long-term safety of antidepressants and that this would be beneficial in engaging practitioners in the intervention. | Enrolment, internalisation, communal specification | Reflective motivation |
|  | Guidelines for using antidepressants | Summarises the NICE guidance on prescribing antidepressants, including for how long they should be prescribed. | Focus groups with health professionals suggest that there is a need for the information around discontinuation in existing guidance to be highlighted and made more accessible. | Individual specification | Psychological capability |
|  | Research on relapse rates | Summary of research on relapse rates in patients who withdrew from antidepressants by taking a placebo. | Think aloud interviews and focus groups suggest that practitioners are fearful of destabilising currently well patients and may need reassuring that many patients will continue to feel well after discontinuation. | Systematisation | Reflective motivation |
|  | Alternatives to antidepressants | Provides evidence that psychological methods can also prevent relapse | Practitioners in the focus groups report that they would like an intervention to be evidence-based and that this would help them to engage with the intervention. | Enrolment | Reflective motivation |
|  | Why reduce and stop antidepressants (2) | Highlights patient experiences with side effects and that psychological support can be helpful in preventing relapse. | Focus groups with health professionals indicate that practitioners are not always aware of reasons why the patient may want to discontinue. |  | Reflective motivation |
| Broaching the subject | Broaching the subject | Summary of module content | Primary qualitative work suggests that practitioners disagree about whether the responsibility for raising the possibility of discontinuation lies with the practitioner or patient. Many agreed that it should be a shared decision. This module therefore highlights this issue to practitioners, along with evidence that many patients feel it is the practitioner’s responsibility to initiate the discussion around withdrawal. | Legitimation, skill-set workability | Social opportunity |
|  | Who should initiate the conversation? | Highlights conflicting views between patients and practitioners about who should raise the issue |  | Skill set workability | Reflective motivation, social opportunity |
|  | The role of the GP | Highlights what patients expect from their GP with regards to managing discontinuation. |  | Skill set workability | Social opportunity |
|  | Patient perspectives | Acknowledge that patients may have understandings of antidepressants that do not facilitate discontinuation (e.g. seeing depression as a life-long condition caused by low serotonin levels) | As there are a number of conflicting ideas about how antidepressants work, and belief in the serotonin hypothesis is considered a barrier to withdrawal for patients (according to the primary qualitative work and the qualitative synthesis), this information was included to ensure all practitioners provide information that is consistent with the information given to patients regarding how antidepressants work. | Communal specification, internalisation | Psychological capability |
|  | Talking to the patient | Highlights the importance of stating clearly that you plan to discuss antidepressant withdrawal. Explains that patients may need reassuring that they can come off and stay well. Agreeing a time to start tapering | Practitioners reported that both time constraints in consultations and confidence to de-prescribe were important in terms of being able to support patients through discontinuation. Providing information on how to have these initial discussions may build confidence and will also help practitioners to manage their limited time. | Legitimation | Physical capability, Psychological capability |
|  | Reassurance and addressing concerns | Ask about patient’s concerns and offer reassurance. Asking about additional concerns earlier in the consultation will help to address these concerns and manage time in consultation | Practitioners reported that both time constraints in consultations and confidence to de-prescribe were important in terms of being able to support patients through discontinuation. Providing information on how to have these initial discussions may build confidence and will also help practitioners to manage their limited time. | Legitimation | Physical capability, Psychological capability |
| When to start tapering | Dealing with relapse | Summary of module content | Practitioners stated during focus groups that an ideal intervention would contain information about when to consider discontinuation and reported that ability to assess the ‘ideal’ patient could be a facilitator to withdrawing. | Internalisation | Psychological capability |
|  | The patient has responded to treatment | Patients who have responded to antidepressants and have few residual symptoms are suitable for tapering |  | Internalisation | Psychological capability |
|  | Things to consider | Those who are currently at high risk of relapse, currently experiencing major life events, do not have adequate support may not be suitable to withdraw. Time of year should also be considered. |  | Internalisation | Psychological capability |
|  | Research on residual symptoms | Evidence that many patients have some residual symptoms and that limiting withdrawal to only those with no symptoms would result in very few patients being offered to discontinue. | Evidence base for consider patients for discontinuation. | Internalisation, | Reflective motivation |
| Antidepressant reduction schedules | Antidepressant reduction schedules | Summarises four plans for withdrawal depending on characteristics of the patient and their treatment | When asked about what information would support them when helping a patient to discontinue ADs, practitioners reported that specific guidance on tapering and information about particular types of ADs is needed. | Internalisation, Activation | Psychological capability |
|  | Plan A | Schedules for patients with few problems, no history of distressing withdrawal and no fear of withdrawal over 4-6 weeks |  | Internalisation, activation | Psychological capability |
|  | Plan B | Schedules for patients taking antidepressants associated with more withdrawal symptoms |  | Internalisation, activation | Psychological capability |
|  | Plan C | Schedules for patients with a difficult history of withdrawal or a fear of withdrawing over 6 weeks or less |  | Internalisation, activation | Psychological capability |
|  | Plan D | Schedules for patients taking tricyclic antidepressants, in particular older patients at risk of cholinergic rebound |  | Internalisation, activation | Psychological capability |
| Dealing with withdrawal symptoms | Withdrawal symptoms (1) | Summary of information in this module | When asked what information needed to be available in the intervention, practitioners reported the need for information around discontinuation effects. |  | Psychological capability |
|  | Withdrawal symptoms (2) | Provides a list of possible symptoms but explains that it may be best to explain to patients only the common symptoms so as to avoid expectations influencing symptoms |  | Individual specification | Psychological capability |
|  | Distinguishing relapse from withdrawal | Information about the differences between withdrawal and relapse |  | Individual specification, differentiation | Psychological capability |
|  | Guidance for dealing with withdrawal symptoms | Guidance on dealing with mild, moderate and severe withdrawal symptoms as well as guidance on dealing with patients who report suicidal thoughts |  | Individual specification | Psychological capability |
| Dealing with relapse | Dealing with relapse | Summary of module content | Practitioners suggested that relapse prevention planning tools should be provided for both practitioners and patients. While patients receive information about how to self-manage through identifying their warning signs and triggers, practitioners are provided with links to this patient information as well as guidance on dealing with and preventing relapses with patients. |  | Psychological capability |
|  | Distinguishing relapse from withdrawal | Information about the differences between relapse and withdrawal |  | Individual specification, differentiation | Psychological capability |
|  | ADvisor to help patients prevent relapse | A summary of how the patient digital intervention, ‘ADvisor’, can support patients in recognising warning signs and managing stress in order to help prevent relapse. |  | Internalisation | Psychological capability |
|  | Treating relapse | A summary of when it might be advisable to reinstate their antidepressants (symptoms of relapse not caused by withdrawal, not helped by relapse prevention techniques and not helped by techniques for dealing with difficult life events) |  | Individual specification | Psychological capability |
| ADvisor for patients |  | This section gives a brief overview of the content in patient intervention. | Practitioners report time constraints as a barrier to managing withdrawal. By allowing practitioners to view and recommend content for patients to look at outside of the consultation, this may help practitioners to support patients within their time constraints. | Communal specification | Physical capability |
| Printable pages |  | This section provides a page that can be printed and given to the patient. | GPs reported that the physical act of handing a patient a piece of paper can have therapeutic value in a consultation. | Differentiation | Physical capability |
| Resources |  | Links to relevant papers and guidelines | Practitioners reported during focus groups that they had difficulty with accessing current guidelines and did not always know where to find these. | Relational integration | Psychological capability |
